# Macrolide-Resistant *Mycoplasma pneumoniae*, United States^1^

**DOI:** 10.3201/eid2108.150273

**Published:** 2015-08

**Authors:** Xiaotian Zheng, Stella Lee, Rangaraj Selvarangan, Xuan Qin, Yi-Wei Tang, Jeffrey Stiles, Tao Hong, Kathleen Todd, Amy E. Ratliff, Donna M. Crabb, Li Xiao, T. Prescott Atkinson, Ken B. Waites

**Affiliations:** Ann & Robert H. Lurie Children’s Hospital of Chicago, Northwestern University Feinberg School of Medicine, Chicago, Illinois, USA (X. Zheng, S. Lee, K. Todd);; The Children's Mercy Hospitals and Clinics, Kansas City, Missouri, USA (R. Selvarangan);; Seattle Children’s Hospital, Seattle, Washington, USA (X. Qin);; Memorial Sloan-Kettering Cancer Center and Weill Medical College of Cornell University, New York, New York, USA (Y.W. Tang, J. Stiles);; Hackensack University Medical Center, Hackensack, New Jersey, USA (T. Hong);; University of Alabama at Birmingham, Birmingham, Alabama, USA (A.E. Ratliff, D.M. Crabb, L. Xiao, T.P. Atkinson, K.B. Waites)

**Keywords:** Mycoplasma pneumoniae, macrolide, antimicrobial resistance, MIC, United States, children, bacteria, United States

## Abstract

Macrolide-resistant *Mycoplasma pneumoniae* (MRMP) is highly prevalent in Asia and is now being reported from Europe. Few data on MRMP are available in the United States. Using genotypic and phenotypic methods, we detected high-level MRMP in 13.2% of 91 *M. pneumoniae*­–positive specimens from 6 US locations.

Macrolides are the preferred treatment for infections caused by *Mycoplasma pneumoniae* in children (*1*). Since 2000, macrolide resistance has developed in Asia and has now been reported from many parts of the world (*2*). This resistance is of a high level and has been associated with longer duration of fever, cough, and hospital stay and the need to switch to alternative antimicrobial agents (*3*–*5*). Scant information is available about the prevalence of macrolide-resistant *M. pneumoniae* (MRMP) in the United States, and no organized ongoing surveillance exists.

## The Study

During August 2012–April 2014, respiratory specimens that tested positive for *M. pneumoniae* by molecular methods were collected from 6 medical centers throughout the United States. These sites were located in Chicago, Illinois; Kansas City, Missouri; Hackensack, New Jersey; New York, New York; Seattle, Washington; and Birmingham, Alabama. Five medical centers tested for *M. pneumoniae* DNA using the FilmArray respiratory pathogen panel (Biofire Diagnostics, Salt Lake City, UT, USA). The University of Alabama at Birmingham used a laboratory-developed real-time PCR targeting *RepMp1*.

Testing for 23S rRNA mutations conferring macrolide resistance was performed on original specimens by real-time PCR melt curve analysis (*6*) and confirmed by DNA sequencing at the Lurie Children’s Hospital (Chicago). Subculture and phenotypic antimicrobial susceptibility testing was performed by using a broth microdilution method approved by the Clinical and Laboratory Standards Institute at the University of Alabama at Birmingham. The study comprised 91 *M. pneumoniae*–positive specimens. Patients’ ages ranged from 10 months to 66 years; 83 (91.2%) samples were from patients <18 years of age. Specimen types were nasopharyngeal or nasal swabs (72 samples), nasal aspirate/washes (12 samples), bronchoalveolar lavage (5 samples), and throat swab and tracheal aspirate samples (1 sample each).

We found a 23S rRNA point mutation A2063G known to confer macrolide resistance in 10 (10.9%) of 91 specimens by direct real-time PCR with melting curve analysis. All mutations were confirmed by DNA sequencing. *M. pneumoniae* grew in subculture from 62 (68.1%) of these specimens. PCR and sequencing on those subcultures confirmed macrolide resistance in 6 specimens that had been previously identified by direct real-time PCR and in 2 additional specimens that had not previously been detected by PCR. The 2 specimens most likely tested negative for mutations by direct PCR because of a low number of organisms in the original sample. Macrolide resistance was not significantly correlated with patient age or specimen type (data not shown). MRMP was detected in a total of 12 (13.2%) samples (Table).

**Table:** Macrolide-resistant *Mycoplasma pneumoniae*–positive respiratory specimens, selected US sites, August 2012–April 2014

Collection site	Specimens	
	No. tested	No. (%) macrolide resistant
Chicago, IL	23	4 (17.4)
Kansas City, MO	40	3 (7.5)
Hackensack, NJ	2	1 (50.0)
New York, NY	5	2 (40.0)
Seattle, WA	15	1 (6.7)
Birmingham, AL	6	1 (16.7)
All	91	12 (13.2)

We conducted phenotypic antimicrobial susceptibility testing on all 62 *M. pneumoniae* isolates obtained by subculture. All 54 macrolide-susceptible isolates showed very low erythromycin MICs (<0.008 μg/mL), whereas the 8 isolates that had the A2063G mutation showed uniformly high erythromycin MICs (>256 μg/mL). All 62 isolates were susceptible to tetracycline (MIC 0.031–1 μg/mL) and levofloxacin (MIC 0.031–1 μg/mL).

The FilmArray respiratory pathogen panel detects multiple respiratory pathogens. Of the 80 respiratory specimens for which co-infection data were available, 26 (32.5%) had a viral pathogen detected along with *M. pneumoniae*. These viruses were 15 (18.8%) rhinovirus/enterovirus group viruses, 3 (3.8%) respiratory syncytial viruses, 4 (5%) parainfluenza viruses, 1 (1.3%) human metapneumoviruses, and 3 (3.8%) coronaviruses.

## Conclusions

MRMP prevalence has been reported to range from 2% to 26% in European countries (*7*,*8*) and is 30% in Israel (*9*). The prevalence is much higher in Asia, where MRMP first emerged in 2000, and now exceeds 90% in some areas of China and Japan (*10*,*11*).

Macrolide resistance has been documented in North America since 2008 (*6*,*12*). The Centers for Disease Control and Prevention recently published results from 199 specimens obtained from case-patients, small clusters, and outbreaks that occurred during 2006–2013 but did not specify geographic locations from which specimens were derived. An overall 10% rate of macrolide resistance was reported (*13*). Yamada et al. tested 49 *M. pneumoniae*–positive specimens collected from children in St. Louis, Missouri, USA, during 2007–2010 and found 4 (8.2%) that contained the A2063G mutation (*14*). Eshaghi et al. detected MRMP in 12.1% specimens collected in Ontario, Canada, during 2010–2011 (*15*). 

Our finding of high-level macrolide resistance in 13.2% of specimens from all 6 centers throughout a broad geographic area in the United States proves this problem has emerged in all regions of the nation and might increase over time, as it has in other countries. The design of our epidemiologic study provides the most accurate estimate of the point prevalence of MRMP available thus far. Previous studies reported from the United States were limited primarily to individual case reports, clusters, outbreaks, or single geographic locations.

The mechanism for macrolide resistance in *M. pneumoniae* is point mutations in a few positions of domain V of the peptidyl transferase loop of 23S rRNA, the location of macrolide binding to the 50S bacterial ribosome subunit (*3*). The A2063G transition mutation has been the most common one detected in most studies and was the only mutation found in our study. Although less common, other mutations conferring macrolide resistance, but not found in our study, include A2063T or C, A2064G, A2067G, and C2617A or G (*3*,*10*,*15*).

Unlike previous studies, our study used both molecular and phenotypic techniques to detect macrolide resistance. Thus, we were able to correlate erythromycin MICs with the presence of 23S rRNA mutations. Our results showed striking differences in erythromycin MICs between the susceptible and resistant isolates and confirmed high-level resistance to erythromycin in every isolate. Erythromycin is usually used for testing because mutations in A2063 and A2064 consistently have been shown to cause high resistance to both erythromycin and azithromycin (*3*,*5*,*10*). A limitation of the study is the nature of anonymous specimen collection. Inclusion of patient information, especially antimicrobial therapy, will enhance data interpretation in prospectively conducted future studies.

One notable observation from our study is the co-infection of *M. pneumoniae* with various viral pathogens. A viral co-infection rate >30% supports the use of multiplex testing for viral and bacterial pathogens in children with respiratory infections of uncertain etiology.

Although our study has confirmed MRMP in 6 geographically diverse US states, macrolides should remain the drugs of choice in children with *M. pneumoniae* respiratory infections. Clinicians should be vigilant for macrolide treatment failures and consider using alternative drugs if necessary. Countries such as Japan and China that have a very high macrolide resistance rate for *M. pneumoniae* and other respiratory pathogens often have high consumption of macrolides. Okada et al. (*5*) reported that macrolides account for 30% of all oral antibacterial drugs in Japan and concluded that the increase in macrolide-resistant bacteria during the past several years in that country was closely related to selective pressure resulting from widespread antimicrobial use. Given the common use of macrolides in the United States to treat pediatric respiratory infections, judicious use of antimicrobial drugs should be emphasized. Reevaluation of existing classes and investigation of new classes of antimicrobial agents may be prudent to have additional treatment alternatives for MRMP infections beyond tetracyclines and fluoroquinolones. Surveillance for this resistance in the United States will help monitor the trend.
